# Efficacy of long-lasting insecticidal nets before and after the selection of pyrethroid-resistant *Anopheles gambiae* s.l. and *Anopheles funestus* s.l. in northeastern Tanzania: A longitudinal meta-analysis of experimental hut trials at a single location

**DOI:** 10.1186/s13071-026-07294-4

**Published:** 2026-02-25

**Authors:** Patrick Kija Tungu, Wema Sudi, Benard Batengana, William Nhandi Kisinza, Mark W. Rowland

**Affiliations:** 1 Amani Medical Research Centre, National Institute for Medical Research, P.O. Box 81, Muheza, Tanzania; 2 Department of Disease Control, London School of Hygiene & Tropical Medicine, London, WC1E 7HT UK; 3 Pan-African Malaria Vector Research Consortium (PAMVERC), P.O. Box 81, Muheza, Tanga, Tanzania

**Keywords:** Long-lasting insecticidal net, Pyrethroid resistance, *Anopheles gambiae*, *Anopheles funestus*, Experimental hut trial, Meta-analysis, Tanzania

## Abstract

**Background:**

The extent to which insecticide resistance is affecting malaria vector control in community and home is not fully understood. This study assessed the implications of insecticide resistance for entomological efficacy of long-lasting insecticidal nets (LLINs) against wild free-flying field *Anopheles gambiae* s.l. and *Anopheles funestus* s.l. in experimental hut trials (EHT) in northeastern Tanzania before and after the evolution of pyrethroid-resistance.

**Methods:**

Evaluations of LLIN efficacy were conducted according to World Health Organization (WHO) guidelines in ten EHT commissioned by WHO between 2006 and 2017, before and after resistance development in Muheza. The entomological parameters taken into account were mortality, blood-feeding inhibition, induced exophily, personal protection, mass killing effect, and deterrence. WHO bioassays determined resistance levels in terms of susceptibility profile and intensity of resistance, while polymerase chain reaction (PCR) molecular diagnostics detected resistance alleles (L1014S and L1014F) and identified mosquitoes to species.

**Results:**

Anopheline mosquitoes were fully susceptible to pyrethroids until 2010, when they began to show resistance. The voltage gated sodium channel (VGSC) L1014S point mutation kdr was detected in *An. gambiae* s.l. at allelic frequency of 47%; no L1014F point mutation was detected. Synergist tests with piperonyl butoxide (PBO) restored efficacy only partially, indicating involvement of metabolic mechanisms. Meta-analysis of the ten EHT showed that mortality of susceptible *An. gambiae* s.l. was 6.7- and 5.2-fold greater on zero-times (*z* = 6.6, *P* = 0.001) and 20-times washed LLINs (*z* = 2.3, *P*= 0.023) than against resistant *An. gambiae* s.l. The mortality of unwashed and washed LLINs against susceptible *An. funestus* s.l. was 3.3-fold (*z* = 2.8, *P* = 0.004) and 2.6 (*z* = 2.9, *P* = 0.004) greater than against resistant *An. funestus* s.l. Resistant Anophelines were more likely to exit the huts as compared with susceptible Anophelines (*z* = 2.79, *P* = 0.005). The transition from susceptibility to resistance on changes to blood-feeding rates was nonsignificant for either species.

**Conclusions:**

Reduced mortality induced by LLINs after selection of pyrethroid resistance indicates that resistance undermines household control of vector populations. Personal protection indicators such as proportions feeding on blood seemed less affected by the transition to resistance. Meta-analysis, comparing the same net brands before and after selection of resistance, revealed the factors (decreases in mortality, status in blood-feeding rates, and increases in pyrethroid-induced exiting rates) most affected by resistance.

**Graphical Abstract:**

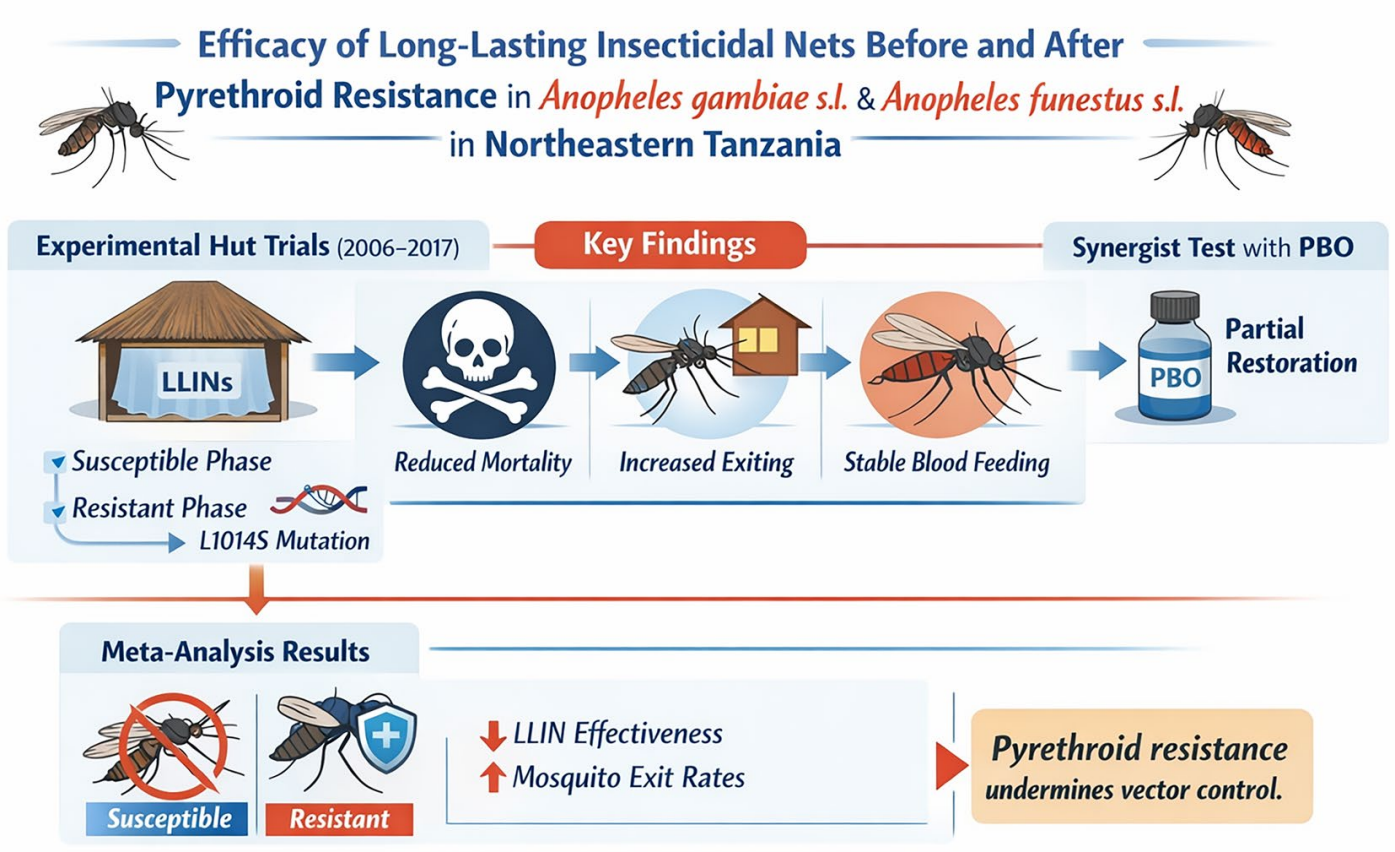

**Supplementary Information:**

The online version contains supplementary material available at 10.1186/s13071-026-07294-4.

## Background

Long-lasting insecticidal nets (LLINs) are the primary tool for malaria vector control in sub-Saharan Africa [1, 2]. The use of LLINs on a large scale not only decreases malaria morbidity and mortality [3] but also provides community protection by reducing the longevity of malaria vector mosquito populations [4].

Over the last 15 years LLINs have been scaled up massively in sub-Saharan Africa [5] initially by targeting pregnant women and children under 5 years of age [6] and latterly by aiming for universal coverage of communities [7, 8].

Ever since the World Health Organization (WHO) approved the use of LLINs as a primary strategy for malaria control [9], long-lasting formulations of pyrethroid, owing to wash-durability, residual activity, and cost-effectiveness have been the primary class of insecticide used on nets [10]. Resistance to pyrethroid insecticide in Anopheline mosquitoes caused by a variety of mechanisms, most notably the target site insensitivity mechanism VGSC *kdr* [11, 12] and metabolic mechanisms caused by mixed function oxidases (MFOs) [13–15] are now firmly established in many African countries as resistance continues to spread [16, 17].

In Tanzania, the spread and causes of pyrethroid resistance were discerned through annual national, sentinel site surveys of insecticide susceptibility [18–20]. However, a high frequency of resistance as indicated by WHO susceptibility tests does not necessarily translate into vector control failure, nor does it necessarily lead to a negative impact of LLINs on vector control [21]. The substantiation of resistance demonstrated by WHO susceptibility tests requires corroboration of impact on vector control in households as measured using other WHO standardized approaches, such as Phase II experimental hut trials (EHT) against wild host-seeking resistant mosquitoes [22], and longitudinal multi-site village observations in which surveillance for increasing malaria transmission and entomological inoculation rate are monitored [23, 24].

The impact of pyrethroid resistance on malaria transmission, and the efficacy of control tools may differ from one location to another [17, 25–32].

Over the period 2006–2017, National Institute for Medical Research (NIMR) and London School of Hygiene and Tropical Medicine (LSHTM) were commissioned by WHO under the auspices of the WHO Pesticide Evaluation Scheme (WHOPES) and the Prequalification Team for Vector Control (WHO/PQT-VC) to undertake a series of LLIN product evaluations according to standardized methodology in suites of verandah-trap experimental huts of “the East African design” in Muheza, Northeast Tanzania [22]. The main objective of the WHO hut trials was to assess efficacies and wash-fastness of individual brands of LLIN before and after standardized washing to determine whether candidate LLIN nets met the efficacy thresholds required by WHO against wild, free-flying mosquitoes under standardized household conditions. This involved recording mosquito entry and exiting into window and verandah traps and recording of host feeding and mosquito mortality. While the EHT site location, Zeneti (Muheza), has not changed over this period, the composition of the mosquito populations may have altered under selection pressure from intermittent campaigns of universal net coverage led by the National Malaria Control Programme (NMCP).

Thus, the NIMR/LSHTM project on LLIN product development and evaluation for WHO provides a unique opportunity to study the evolution of insecticide resistance over the decade under natural household conditions, and its effect on behavior and survival of *An. gambiae* s.l. and *An. funestus* s.l.

There have been other multi-site studies that have been helpful in determining possible associations between resistance and LLIN efficacy at a given point in time. However, these studies cannot substantiate a causal relationship between changes in LLIN efficacy and changes in resistance. The current study that uses data from a single location with the fixed hut and the shifting status of the vector resistance offered a special chance to examine the shifting effectiveness of standard LLINs and their ability to manage mosquitoes at a location where the vector population had grown increasingly resistant over the past 10 years.

## Methods

### Study area and experimental huts

The experimental hut trials were conducted at the NIMR field station at Zeneti village 5° 13′ S latitude, 38° 39′ E longitude, and 193 m altitude, where *An. gambiae* s.l. and *An. funestus* s.l. were the major malaria vectors [33], with entomological inoculation rates (EIRs) varying between 34–405 infective bites per person per year at different times [34]. During the trials between 2006 and 2010 the local population of *An. gambiae* s.l. were 100% susceptible to pyrethroids [35, 36]. During the hut trials conducted between 2011 and 2013, the vector species have become resistant to pyrethroid [19].

The huts were constructed to a design described by WHO [37], based on the original verandah hut developed in Tanzania but with significant structural modifications [38, 39]. These included reduction of the eave gaps to 2 cm, introduction of wooden, hessian-lined ceilings, roofs of corrugated iron, concrete floors surrounded by water-filled moats, and unidirectional baffles over eave gaps to funnel host-seeking mosquitoes into the rooms [40, 41]. The huts had open eaves with verandah traps and window traps on each side. The working principle of these huts has been described by WHO in 2013 [36]. Each day of the trial, two opposite sides of the huts had window traps and closed verandahs that were screened to capture mosquitoes leaving via the eaves. The other two verandahs were left unscreened so that mosquitoes entering through the eaves were restricted from exiting by the eave baffles [40, 41].

While most experimental hut trials were premeditated and commissioned by WHO, the meta-analysis was opportunistic and took advantage of the evolution of resistance to open a window to explore what resistance might mean for vector control. The brands of LLIN assessed over the 10 years are listed in Table 1. All products were approved by WHO. Some LLIN were made of polyethylene, others of polyester. Some were coated with the active ingredient in formulated resin, and others had the active ingredient incorporated in the fiber during manufacture. All contained pyrethroid as the active ingredient at different concentrations, some contained deltamethrin, and others contained permethrin, alpha-cypermethrin, or lambda-cyhalothrin. The original aim was to test LLIN capacity to meet the WHO efficacy criteria after 20 washes. All those cited in Table 1 met one or more WHO criteria.

**Table 1:** Description of trials and treatment arms^*^

Serial number	Year of trial	LLIN product	AI concentration/m^2^	Netting material	Wash status of arms	Status of local
1	2006	Interceptor™	Alpha-cypermethrin 200 mg/m^2^	Polyester	Unwashed and 20 times washed	Susceptible
2	2008	DuraNet	Alpha-cypermethrin 261 mg/m^2^	Polyethylene	Unwashed and 20 times washed	Susceptible
3	2008	PermaNet 2.0	Deltamethrin 55 mg/m^2^	Polyester	Unwashed and 20 times washed	Susceptible
4	2010	Olyset Net	Permethrin 2000 mg/m^2^	Polyethylene	Unwashed and 20 times washed	Susceptible
5	2013	Olyset Net	Permethrin 2000 mg/m^2^	Polyethylene	Unwashed only	Resistant
6	2013	Interceptor™	Alpha-cypermethrin 200 mg/m^2^	Polyester	Unwashed only	Resistant
7	2013	PermaNet 2.0	Deltamethrin 55 mg/m^2^	Polyester	Unwashed and 20 times washed	Resistant
8	2014	PermaNet 2.0	Deltamethrin 55 mg/m^2^	Polyester	Unwashed and 20 times washed	Resistant
9	2015	Olyset Net	Permethrin 2000 mg/m^2^	Polyethylene	Unwashed only	Resistant
10	2017	Interceptor™	Alpha-cypermethrin 200 mg/m^2^	Polyester	Unwashed and 20 times washed	Resistant

AI, active ingredient

^*^All these studies were conducted in Muheza, Tanzania, between 2006 and 2018 and formerly published in Reports of the WHOPES Working Group Meetings or on the WHO Pre-qualification Team (PQT) website

### Net preparation and washing

The long-lasting insecticidal nets were washed according to WHOPES Phase II protocols [37]. Each net was washed individually in 10 L of tap water containing 2 g/L soap solution (“Savon de Marseille”), subjected to 20 rotations per minute for 6 min during 10-min immersion, and then twice rinsed.

The interval between washes was in accordance with the regeneration time of the candidate net, as predetermined by WHO. The WHO-predetermined regeneration time was confirmed by pre-trial assays conducted using one insecticide-treated net (ITN) from each study arm using susceptible colony of *An. gambiae* (Kisumu).

The washing schedule used a stepped design to ensure that final washes of all treatment arms were completed on the same day. To simulate wear and tear a total of six 4 cm × 4 cm holes were made in each net (two holes cut on each side and one hole on each end).

### Experimental hut trial design

The ten experimental hut trials included in the study were conducted between 2006 and 2017 and employed standard LLIN treatment arms and the same two suites of huts. Each trial employed the same basic study design in which Latin square rotation adjusted for any variation between hut position, individual volunteer sleeper attractiveness, and individual net. All ten hut trials had at least the first three arms and usually four arms, the fourth being a positive control, that was analyzed comparatively in the study: (i) unwashed LLIN (0W), (ii) LLIN washed 20 times (20W), (iii) untreated unwashed polyester net (0W) (negative control), and (iv) hand-dipped ITN washed until just before the threshold 80% mortality in cone bioassay (positive control). The details of the trials and treatment arms are listed in Table 1.

The primary outcomes compared between trial arms were (i) deterrence, (ii) treatment-induced exiting, (iii) mortality, (iv) overall killing effect, (v) blood-feeding inhibition, and (vi) personal protection. The first, deterrence, refers to the reduction in entry into treatment huts relative to the control huts (i.e., those containing untreated nets); this was estimated by the calculation *deterrence (%)* = *100((Tc − Tt)/Tc)*, where *Tc* was the total mosquitoes entering untreated control huts, and *Tt* was the total mosquitoes entering huts fitted with treated net. Second, treatment-induced exiting refers to the proportion of mosquitoes found in exit traps of treatment huts relative to the same proportion in control huts; this was estimated by the calculation *exophily (%)* = *100((tv* + *tex)/T)*, where *tv* was the total mosquitoes collected from huts verandah, *tex* was the total mosquitoes collected from huts exit traps, and *T* was the total entering the huts. Third, mortality is the proportion of mosquitoes killed relative to the total catch size as derived from the formula *mortality (%)* = *100(Td/T)*, where *Td* was the number dead in the huts, and *T* was the total entering the huts. Fourth, the overall killing effect refers to the numbers killed by a treatment relative to the untreated control, as derived from the formula *killing effect (%)* = *100(Kt − Ku)/Tu*, where *Kt* was the number killed in the huts with treated nets, *Ku* was the number dead in the huts with untreated nets, and *Tu* was the total entering the huts with untreated nets. Fifth, blood-feeding inhibition is the proportional reduction in blood feeding in huts with treated nets relative to controls with untreated nets; blood-feeding inhibition (%) was calculated as follows: *((Bfu − Bft)/Bfu) × 100*; here, *Bfu* is the proportion of blood-fed mosquitoes in the untreated control huts, and *Bft* is the proportion of blood-fed mosquitoes in the huts with a specific insecticide treatment. Sixth, personal protection is the reduction in mosquito biting owing to treated nets relative to untreated nets, which was derived from the formula *% Personal protection* = *100(Bu-Bt)/Bu*, where *Bu* was the total number blood-fed mosquitoes in the huts with untreated nets, and *Bt* was the total number blood-fed in the huts with treated nets.

Treatment arms were rotated once or twice through each hut according to Latin square designs. A treatment was assigned at random to a particular hut for 6 nights’ observation before being transferred to the next hut. Male volunteers slept on beds under the nets between the hours of 7:30 p.m. and 6:30 a.m. The sleepers were rotated through the huts on consecutive nights. Six nets were available per treatment arm, and each net was tested on consecutive nights during the 6-night rotation. At the end of the weekly rotation the huts were cleaned and aired for 1 day before starting the next rotation. Each morning dead and live mosquitoes were collected from the verandahs, room, and window traps. Live mosquitoes were provided with 10% sugar solution. Delayed mortality was recorded after 24 h. Mosquitoes were identified to species, and gonotrophic status as unfed, blood-fed, semi-gravid, or gravid. Samples of *An. gambiae* s.l. were identified to species by polymerase chain reaction (PCR) [42].

### WHO insecticide susceptibility tests

During each of the trials, susceptibility tests were carried out using WHO test kits for adult mosquitoes [43–45] lined with test papers impregnated with 0.75% permethrin, 0.05% deltamethrin, or 0.05% alpha-cypermethrin. The quality of the test paper was checked against the laboratory susceptible *An. gambiae* s.s. Kisumu strain. Mosquitoes used in these tests were the 2–5-day-old female F1 progeny of the mosquitoes that were collected from Zeneti during each trial. The testing procedure was done according to WHO protocols [43–45].

The resistance status was evaluated on the basis of the WHO criteria, i.e., 98–100% mortality indicates susceptibility; 90–97% mortality indicates possible resistance, and less than 90% mortality indicates resistance. When the control mortality was recorded between 5% and 20%, the mean observed mortality was corrected using Abbott’s formula.

Piperonyl butoxide (PBO) synergy tests were conducted on mosquitoes found to be resistant to permethrin or deltamethrin according to the WHO protocol [45]. The aim of this test was to determine the involvement of mixed function oxidases in the observed phenotypic resistance. In this test, 2–5-day-old F1 adult mosquitoes were pre-exposed to 4% piperonyl butoxide (PBO) test paper for 1 h and then exposed to 0.75% permethrin or 0.05% deltamethrin for 1 h.

### Molecular species identification of the *Anopheles gambiae* complex and presence of knockdown resistance (*kdr*) alleles

*An. gambiae* sibling species identification was carried out according to the standard polymerase chain reaction (PCR) method [42]. Five oligonucleotide primers, GA, ME, AR, QD, and UN, designed from the DNA sequences of the intergenic spacer region of complex ribosomal DNA (rDNA) were used to amplify species-specific DNA sequences to distinguish the members of the *gambiae complex*.

The Taqman assay technique of Bass [46] was used for the detection of the VGSC L1014S and L1014F *kdr* alleles. In some samples, detection of *kdr* alleles were done using conventional PCR method. All *An. gambiae* s.l. mosquitoes collected from huts were analyzed for L1014S or L1014F *kdr* alleles.

### Statistical analysis

Interpretation of the study data required use of several analytical approaches. Meta-analysis was important for demonstrating the differences in pooled efficacy (as measured by the pooled risk ratio of measured parameters) between susceptible and resistant mosquitoes. The generalized linear mixed model was important for determining the significance of differences between treatment arms. Logistic regression was necessary to determine the significance of the association between the change in the efficacy of the tested LLINs and the change in the resistance status of the mosquitoes.

The initial objective, and first analysis, was to compare the efficacy of each LLIN product when zero washed and 20-times washed (trials done between 2006–2017) as per standard WHO Phase II criteria for LLIN analysis [22, 37]. Generalized linear mixed effects logistic regression (with hut, sleeper, and treatment being fixed effects and the time of trial being a random effect) was used to analyze proportional outcomes of treatments: proportions killed (mortality), proportions feeding on blood (or inhibited from feeding), proportions exiting (exophily). Negative binomial regression was used to analyze counts of mosquitoes entering the huts (deterrence), blood feeding (personal protection), or dying (proxy for possible mass killing effect), after adjusting for clustering by day and for variation between sleepers and hut position.

The second objective was to compare bio-efficacy and behavior before and after development of pyrethroid resistance using meta-analysis methods. In the meta-analyses, risk ratios of the proportions dying (mortality), blood feeding, and exiting were pooled using a random-effects meta-analysis model STATA*®* statistical software version 16 (Stata corporation, College Station, Texas 77845 USA, 2019) before and after the development of resistance. Overall heterogeneity across trials was calculated using Cochrane’s *Q* test with *P* < 0.05 to indicate statistical heterogeneity and quantified heterogeneity using the *I*^2^ statistics.

## Results

### *An. gambiae* s.l. and *An. funestus* s.l. susceptibility tests

**Fig. 1:**
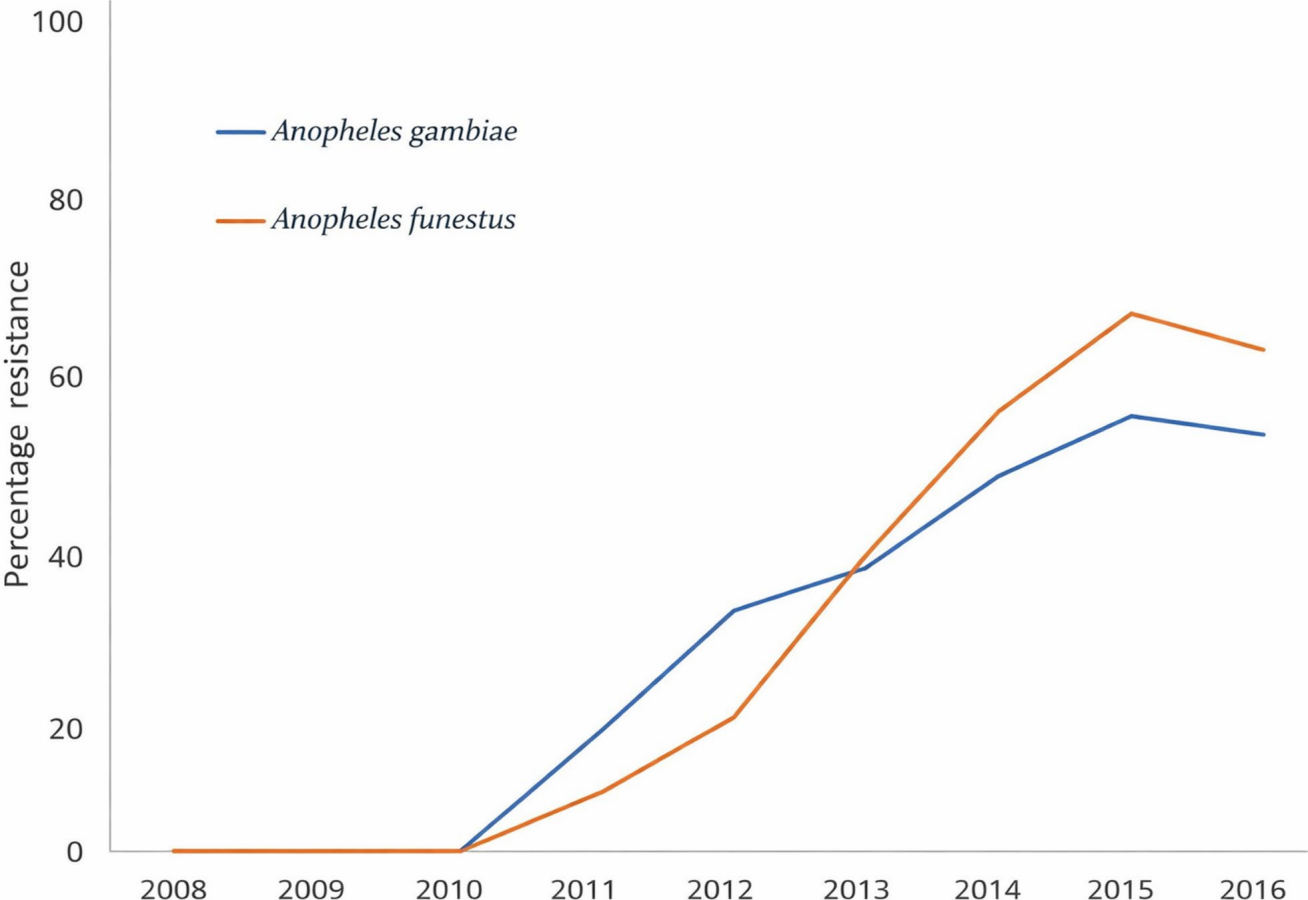
Zeneti field *An. gambiae* s.l. and *An. funestus* s.l. population (2008–2017)—permethrin resistance trend

From 2006 to 2010, WHO resistance tests using 0.05% deltamethrin, 0.75% permethrin, and 0.05% alpha-cypermethrin test papers were conducted on adult mosquitoes collected from huts. These tests showed 100% mortality indicating that *An. gambiae* s.l. was fully susceptible to pyrethroids. Thereafter, in 2013, 2014, and 2015, observing WHO guidance, susceptibility tests using 0.05% deltamethrin were conducted on F1 progeny of *An. gambiae* s.l. collected during the trials from huts with treated and untreated nets; these tests produced reduced mortality of 73%, 71.1%, and 61.3% on test papers (Fig. 1). Susceptibility tests with 0.05% deltamethrin, 0.75% permethrin, and 0.05% alpha-cypermethrin test papers were conducted on F1 of *An. gambiae* s.l. collected from the trial in 2015 from both treated and untreated huts; these tests recorded mortality of 61%, 45%, and 50%, respectively. Similar trends in resistance were observed with *An. funestus* s.l. (Fig. 1).

With both permethrin and deltamethrin, PBO restored efficacy (> 97% mortality) in all *An. gambiae* s.l. and *An. funestus* s.l. mosquitoes tested. This observation suggests that partially, in the case of *An. gambiae*, or fully, in the case of *An. funestus*, metabolic resistance may have contributed to the observed phenotypic resistance to pyrethroids.

### Molecular characterization of mosquitoes.

A total of 1122 mosquitoes collected from the huts during trials were identified using PCR, 94% of the analyzed *An. gambiae* s.l. mosquitoes were *An. gambiae* s.s., and 6% were *An. arabiensis.* All analyzed *An. funestus* s.l. were identified as *An. funestus* s.s.

A total of 833 *An. gambiae* s.s. and 40 *An. arabiensis* were analyzed for VGSC L1014S and L1014F *kdr* allele. All 40 *An. arabiensis* were homozygous susceptible. Of 833 *An. gambiae* s.s., 252 (30%) were homozygous susceptible, 387 (47%) were heterozygous resistant with VGSC L1014S, and 194 (23%) were homozygous resistant for VGSC L1014S. Zero mosquitoes with the VGSC L1014F mutation were detected.

### Experimental hut trials

#### Mosquito mortality and overall killing effect

The proportions killed with the LLIN treatments and dying in the untreated control are shown in Tables 2–4. For the deltamethrin-based LLIN, PermaNet 2.0, experimental hut trials were conducted in 2008, 2013, and 2014. In these trials, all insecticide-treated nets recorded significantly greater mortality against *An. gambiae* s.l. than were recorded with untreated control nets (Table 2). In the 2008 trial, when *An. gambiae* s.l. was fully susceptible, the unwashed PermaNet 2.0 recorded significantly greater mortality than was recorded with PermaNet 2.0 washed 20 times and conventionally treated net (CTN) washed three times (94.7%, 84.7%, and 68.7% control-corrected mortality [ccm], respectively). During the 2013 trial when *An. gambiae* s.l. had become resistant, the unwashed PermaNet 2.0, PermaNet 2.0 washed 20 times, and CTN washed 3 times recorded reduced mortality against *An. gambiae* s.l. (23.9%, 21.6%, and 18.3% ccm) compared with 2008. A year later, in the 2014 trial, percentage mortality against *An. gambiae* s.l. across all three treatments never reached above 20% (Table 2).

**Table 2:** Experimental hut trials: percentage mortality corrected for control, overall killing effect, blood feeding, blood-feeding inhibition, and personal protection of wild *An. gambiae* s.l. huts during PermaNet 2.0 trials conducted at Zeneti in 2008, 2013, and 2014

		Untreated net	PermaNet^®^ 2.0	PermaNet^®^ 2.0	CTN
Initial dose of deltamethrin (mg/m^2^)		0			25
Number of washes		0	Unwashed	20	3
*Anopheles gambiae*					
Percentage (%) mortality corrected for control	2008	—^a,1^	94.7^b,1^	84.7^c,1^	68.7^d,1^
	2013	—^a,1^	23.9^b,2^	21.6^b,2^	18.3^b,2^
	2014	—^a,1^	6.8^b,3^	0^a,3^	0^a,3^
Percentage (%) overall Killing Effect	2008	—^a,1^	60.9^b,1^	55.6^b,1^	41.9^b,1^
	2013	—^a,1^	12.4^a,2^	26.3^b,2^	16.8^b,2^
	2014	—^a,1^	24^a,2^	0^a,2^	3.4^a,3^
Percentage (%) blood fed (95% CI)	2008	27.9^a,1^	10.3^b,1^	9.2^b,1^	10.5^b,1^
	2013	25.8^a,2^	5.9^b,2^	12.4^c,2^	12.8^c,2^
	2014	34.5^a,2^	14.8^b,3^	30.8^c,3^	27.4^c,3^
Percentage (%) blood-feeding inhibition	2008	—^a,1^	63.21^b,1^	67.02^b,1^	62.29^b,1^
	2013	-^a,2^	77.2^b,2^	51.9^c,2^	50.4^c,2^
	2014	—^a,2^	57.1^b,3^	10.7^a,c,3^	20.6^c,3^
Percentage (%) personal protection	2008	—^a,1^	70.8^b,1^	73.3^b,1^	70.8^b,1^
	2013	—^a,1^	85.2^b,1^	40.9^c,2^	49.6^c,2^
	2014	—^a,1^	10^a,1^	0^a,3^	0^a,3^

Percentage mortality, overall killing effect, blood fed, blood-feeding inhibition, personal protection, and 95% CIs are back-transformed from values calculated by the blocked logistic regression model. Within each column, years not sharing a superscript number differ significantly by blocked logistic regression (*P* < 0.05). Within each row, treatments not sharing a superscript letter differ significantly by blocked logistic regression (*P* < 0.05). CI, confidence interval

The overall killing effect against *An. gambiae* s.l., recorded during the 2008, 2013, and 2014 trials, mirrored the percentage mortality trends (Table 2).

For the permethrin LLIN Olyset Net experimental hut trials were conducted in 2010, 2013, and 2015. All insecticide treatments recorded significantly greater mortality against *An. gambiae* s.l. than against the untreated control (Table 3). With the permethrin LLIN, the mortality recorded against the unwashed and 20-times washed Olyset Nets (100% and 71.9% ccm) was significantly greater than in the 2010 trial, when *gambiae* s.l. was susceptible, than was recorded with Olyset Net during the 2013 and 2015 trials, when the vector was resistant (Table 3). However, the overall killing effect of the unwashed Olyset Net was not significantly greater during the 2010 trial than the killing effects recorded during trials in 2013 and 2015, possibly due to the repellency of permethrin (Table 3).

**Table 3:** Experimental hut trials: percentage mortality corrected for control, overall killing effect, blood feeding, blood-feeding inhibition, and personal protection of wild *An. gambiae* s.l. huts during Olyset Net trials conducted at Zeneti in 2010, 2013, and 2015

		Untreated net	Olyset Net	Olyset Net	CTN
Initial dose of permethrin (mg/m^2^)		0			40
Number of washes		0	Unwashed	20	3
*Anopheles gambiae*					
Percentage (%) mortality corrected for control	2010	0^a,1^	100^b,1^	71.9^c,1^	76.4^c,1^
	2013	0^a,1^	5.1^b,2^	—	3^a,b,2^
	2015	0^a,1^	12.1^a,2^	—	—
Percentage (%) overall killing effect	2010	0^a,1^	10.3^b,1^	38.2^b,c,1^	69.1^c,1^
	2013	0^a,1^	5.9^b,1^	—	5^b,2^
	2015	0^a,1^	5.2^b,1^	—	—
Percentage (%) blood fed (95% CI)	2010	72^a,1^	0^b,1^	9^c^	12^d,1^
	2013	25.8^a,2^	5.3^b,2^	—	12.4^c,1^
	2015	24.1^a,2^	11.1^b,2^	—	—
Percentage (%) blood-feeding inhibition	2010	0^a^	100^b,1^	87.5^c^	83.3^d,1^
	2013	0^a^	79.4^b,2^	—	51.9^b,2^
	2015	0^a^	54.3^b,2^	—	—
Percentage (%) personal protection	2010	0^a^	100^b,1^	92^c^	73.5^d,1^
	2013	0^a^	77.2^b,2^	—	38.6^c,2^
	2015	0^a^	71.4^b,2^	—	—

Percentage mortality, overall killing effect, blood fed, blood-feeding inhibition, personal protection, and 95% CIs are back-transformed from values calculated by the blocked logistic regression model. Within each column, years not sharing a superscript number differ significantly using blocked logistic regression (*P* < 0.05). Within each row, treatments not sharing a superscript letter differ significantly using blocked logistic regression (*P* < 0.05). CI, confidence interval

For the alpha-cypermethrin LLIN (Table 4), Interceptor LN, in the 2006 and 2008 trials when *An. gambiae* s.l. was susceptible, the unwashed Interceptor LN arms recorded significantly greater mortality (91.9% and 96.2%, respectively) than Interceptor LN washed 20 times (76.2 and 83.1, respectively), indicating some loss of active ingredients (AIs) to washing. The mortality recorded with the unwashed and 20-times washed Interceptor LN against *An. gambiae* s.l. in 2006 (91.9% and 76.2% ccm) and in 2008 (96.2% and 83.1% ccm) when *An. gambiae* s.l. was susceptible was significantly greater than the mortality recorded with the unwashed Interceptor LN during the 2013 trial (5.1%) when the vector was resistant (Table 4). Results on the killing effect of the unwashed Interceptor LN mirrored the percentage mortality before and after selection of pyrethroid resistance (Table 4).

**Table 4:** Experimental hut trials: percentage mortality corrected for control, overall killing effect, blood feeding, blood-feeding inhibition, and personal protection of wild *An. gambiae* s.l. huts during Interceptor and DuraNet trials conducted at Zeneti in 2006, 2008, and 2013

		Untreated net	Interceptor	Interceptor	CTN
Initial dose of alpha-cypermethrin (mg/m^2^)		0			40
Number of washes		0	Unwashed	20	3
*Anopheles gambiae*					
Percentage (%) mortality corrected for control	2006	0^a,1^	91.9^b,1^	76.2^c,1^	—
	2008	0^a,1^	96.2^b,1^	83.1^c,1^	65.2^d,1^
	2013	0^a,1^	5.1^b,2^	—	3^b,2^
Percentage (%) overall killing effect	2006	0^a,1^	70.4^b,1^	52.2^c,1^	—
	2008	0^a,1^	69.2^b,1^	60.8^b,1^	53.1^b,c^
	2013	0^a,1^	6.3^b,2^	—	5^b,2^
Percentage (%) blood fed (95% CI)	2006	32.1^a,1^	16.2^b,1^	16.1^b,1^	—
	2008	19.6^a,2^	7.1^b,2^	5.4^b,2^	11.3^b,2^
	2013	25.8^a,2^	7.8^b,2^	—	12.4^c,2^
Percentage (%) blood-feeding inhibition	2006	0^a^	73.8^b,1^	65.8^b,1^	—
	2008	0^a^	63.5^b,1^	72.6^b,1^	42.3^b,1^
	2013	0	69.9^b,1^	—	51.9^b,1^
Percentage (%) personal protection	2006	0^a^	79.5^b,1^	75.6^b,1^	—
	2008	0^a^	71.4^b,1^	78.6^b,1^	50^b,1^
	2013	0^a^	64.9^b,1^	—	38.6^b,1^

Percentage mortality, overall killing effect, blood fed, blood-feeding inhibition, personal protection, and 95% CIs are back-transformed from values calculated by the blocked logistic regression model. Within each column, years not sharing a superscript number differ significantly using blocked logistic regression (*P* < 0.05). Within each row, treatments not sharing a superscript letter differ significantly using blocked logistic regression (*P* < 0.05). CI, confidence interval

#### Meta-analysis pooled estimate of mortality risk

**Fig. 2:**
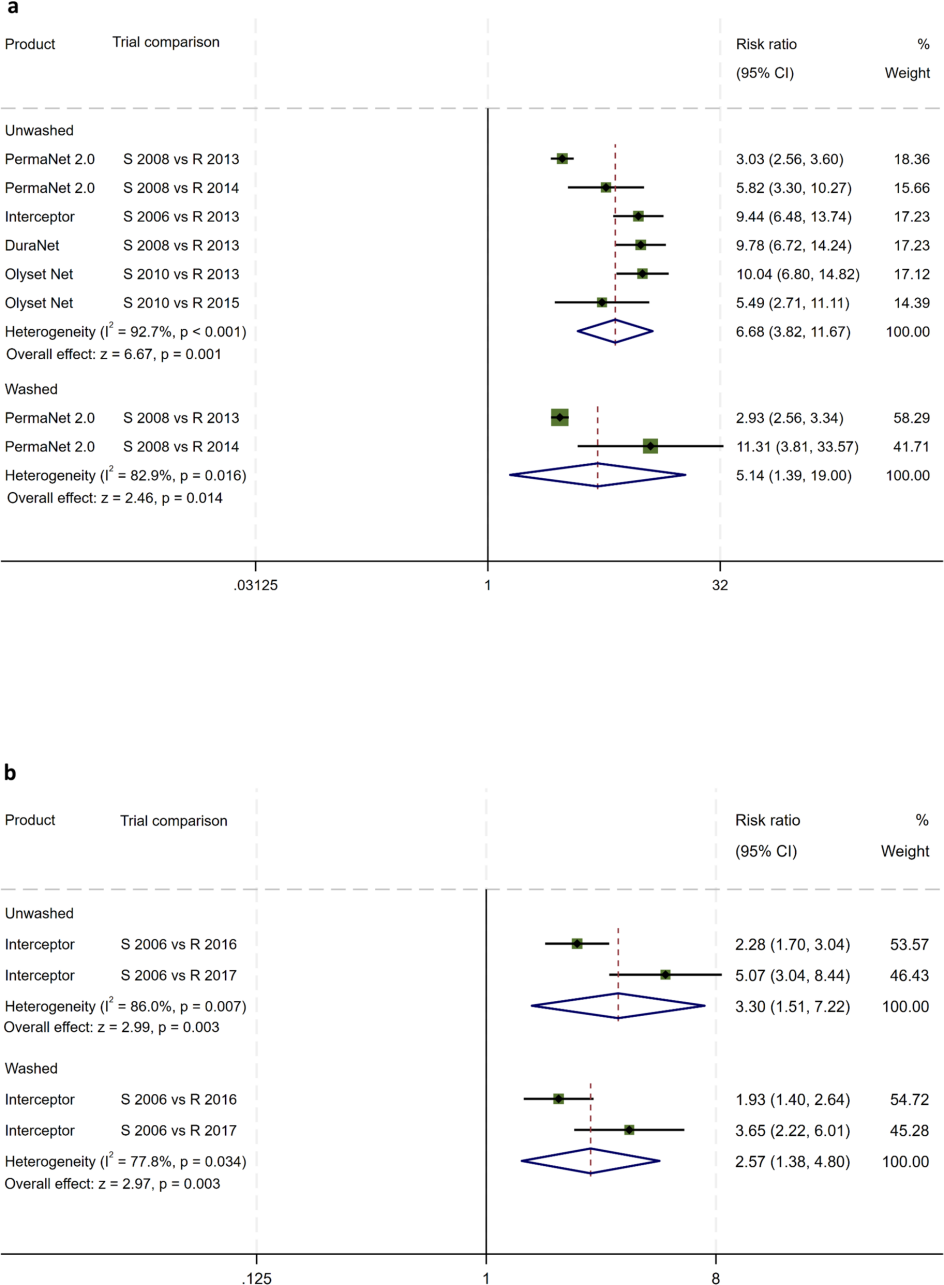
**a** Meta-analysis of the efficacy of washed and unwashed LLIN in hut trials when vectors were susceptible and when resistant: *An. gambiae* s.l.: mortality risk ratios. **b** Meta-analysis of the efficacy of washed and unwashed LLIN in hut trials when vectors were susceptible and when resistant: *An. funestus* s.l.: mortality risk ratios

Meta-analysis across the ten EHT trials showed that, with unwashed LLIN, the pooled relative risk (RR) for mortality of susceptible versus resistant *An. gambiae* s.l. was 6.68-fold (3.81–11.70) greater (*z* = 6.64, *P* = 0.001) (Fig. 2a). With the washed LLIN, the pooled mortality relative risk between susceptible and resistant *An. gambiae* s.l. was 5.23 (1.26–21.76) indicating the mortality ratio induced in the washed LLINs was lower with resistant as compared with susceptible *An. gambiae* s.l. (*z* = 2.26, *P* = 0.023) (Fig. 2a).

The pooled estimate of relative risk for mortality of wild free-flying susceptible versus resistant *An. funestus* s.l. was 3.31-fold (1.45–7.54) with unwashed LLIN and 2.58-fold (1.36–4.90) with washed LLIN. The differences in mortality risk between susceptible to resistant *An. funestus* s.l. was significant in both unwashed (*z* = 2.85, *P* = 0.04) and 20-times-washed LLINs (*z* = 2.89, *P* = 0.004) (Fig. 2b). Washing the LLIN did not lessen their effectiveness against resistant *An. funestus* s.l. as compared with susceptible *An. funestus* s.l. (Fig. 2b).

#### Logistic regression analysis of mortality

**Fig. 3:**
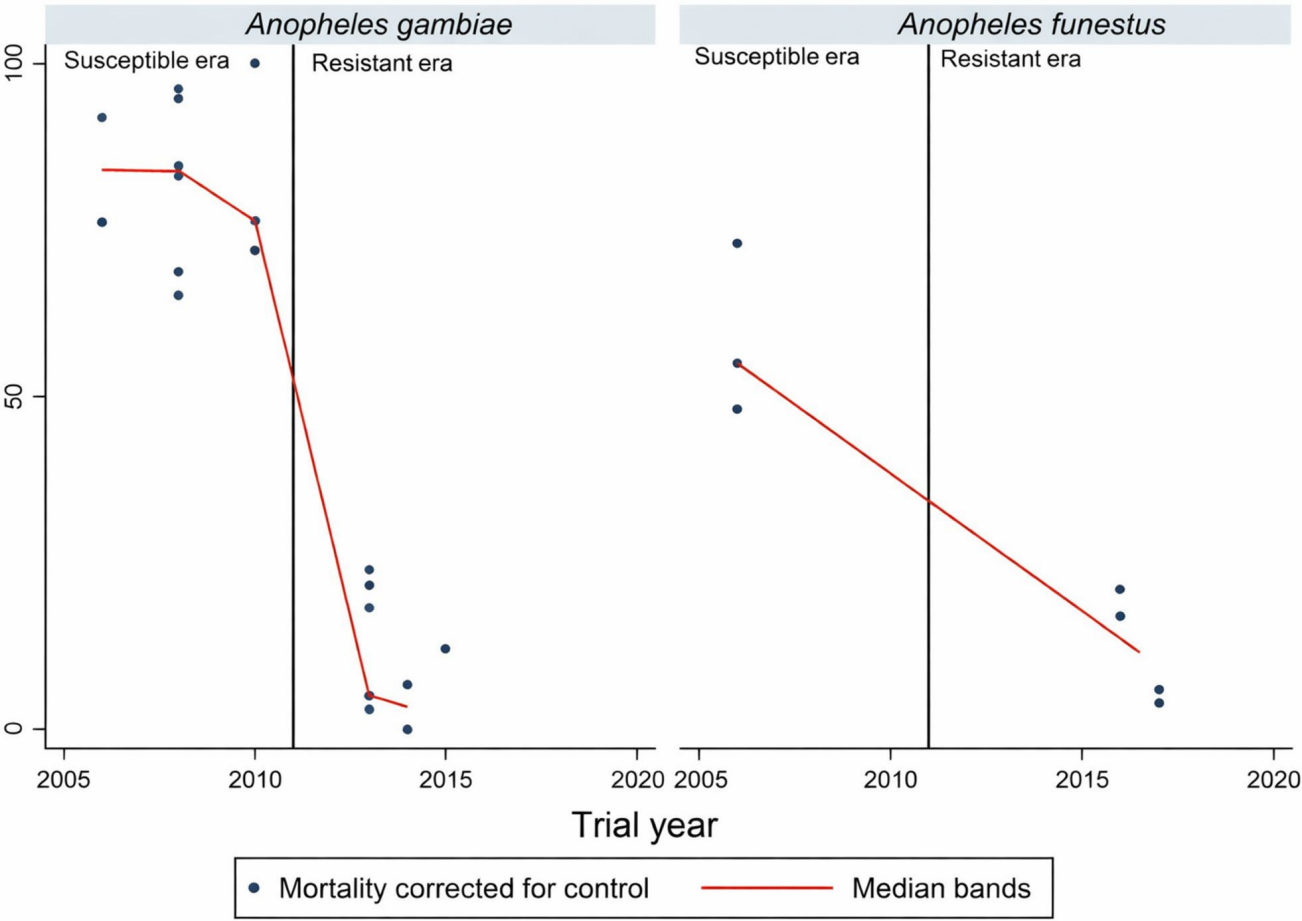
Percentage control-corrected mortality of wild free-flying *An. gambiae* s.l. and *An. funestus* s.l. entering into experimental huts during trials. Each point denotes the mean mortality for each trial. The line graph shows trends of median values for the control-corrected mortalities in various trials. Vertical lines demarcate period of transition from susceptibility to resistance

While the meta-analysis compared mortality risk to mosquitoes between specific LLIN products in relation to the transition from susceptibility to resistance, the logistic regression analysis showed a broad temporal trend over the 10 years, irrespective of LLIN product, spanning the transition from susceptibility to resistance. The trend shows there was significant association between insecticide resistance in *An. gambiae* s.l. and the decline in killing effect of LLIN (*t* = −9.5, *P* = 0.001) (Fig. 3). With *An. funestus* s.l., there was significant association between resistance in *An. funestus* s.l. and the decline in killing effect of LLIN (*t* = −6.65, *P* = 0.001) (Fig. 3).

### Mosquito blood feeding (proportions feeding, blood-feeding inhibition [BFI], and personal protection)

With PermaNet 2.0 in all trials (2008, 2013, and 2014), all three insecticide treatments (unwashed PermaNet 2.0, PermaNet 2.0 washed 20 times, deltamethrin CTN) recorded significantly reduced blood-feeding rates as compared with the untreated control (Table 2).

In 2008 when vectors were susceptible, the unwashed (0 times washed), 20 times washed, and the CTN washed three times recorded a similar percentage of *An. gambiae* feeding on blood (10.3%, 9.2%, and 10.5% as compared with 27.9% in the untreated net). During the 2013 trial when vectors were resistant, unwashed PermaNet 2.0 also recorded significantly lower percentage feeding on blood (5.9%) compared with PermaNet 2.0 washed 20 times and CTN washed 3 times (12.4% and 12.8%, respectively). In the 2014 trial, when *An. gambiae* was highly resistant, the unwashed PermaNet 2.0 also recorded significantly lower percentage feeding on blood (14.8%) compared with PermaNet 2.0 washed 20 times and CTN washed 3 times (30.8% and 27.4%, respectively). The last two were similar statistically (Table 2).

Comparing the 20-times-washed PermaNet 2.0 in 2008, 2013, and 2014, the BFI was 67% when susceptible and 52% when moderately resistant but only 11% when highly resistant in 2014. The BFI in the three-times washed CTN also fell during this period (62% in 2008, 50% in 2013, and 21% in 2014) (Table 2).

Consistent with the BFI results, there was a decrease in personal protection recorded across the three trials over this period (2008, 2013, and 2014) (Table 2). Personal protection was 70.8% and above when the *An. gambiae* population was susceptible, whether PermaNet 2.0 was unwashed or 20-times washed. But when the *An. gambiae* became resistant in 2013, personal protection decreased to 40.9% when 20 times washed and to 0% in 2014 when the population was more resistant (Table 2).

With the Olyset Net trials (2010, 2013, and 2015), all three insecticide treatments (unwashed, washed 20 times, washed CTN 3 times) recorded significantly lower percentage feeding rates as compared with the untreated control (Table 3). Percentage feeding on blood recorded during the 2010 trial (0%), when vectors were susceptible, was lower than that recorded in 2013 and 2015 when resistant (5.3% and 11.1%, respectively); the percentage feeding on blood between trials in 2013 and 2015 was statistically similar. In Olyset Net trials, trends of blood-feeding inhibition recorded mirrored that of personal protection (Table 3).

With the Interceptor LN trials (2006, 2008, and 2013), the three treatments also recorded significantly lower percentage feeding on blood as compared with the untreated control (Table 4). In the comparison between trials, percentage feeding on blood recorded between trials (2006, 2008, and 2013 trials) were also statistically similar. The trend in blood-feeding inhibition mirrored that of personal protection (Table 4).

#### Meta-analysis pooled estimate of blood feeding

**Fig. 4:**
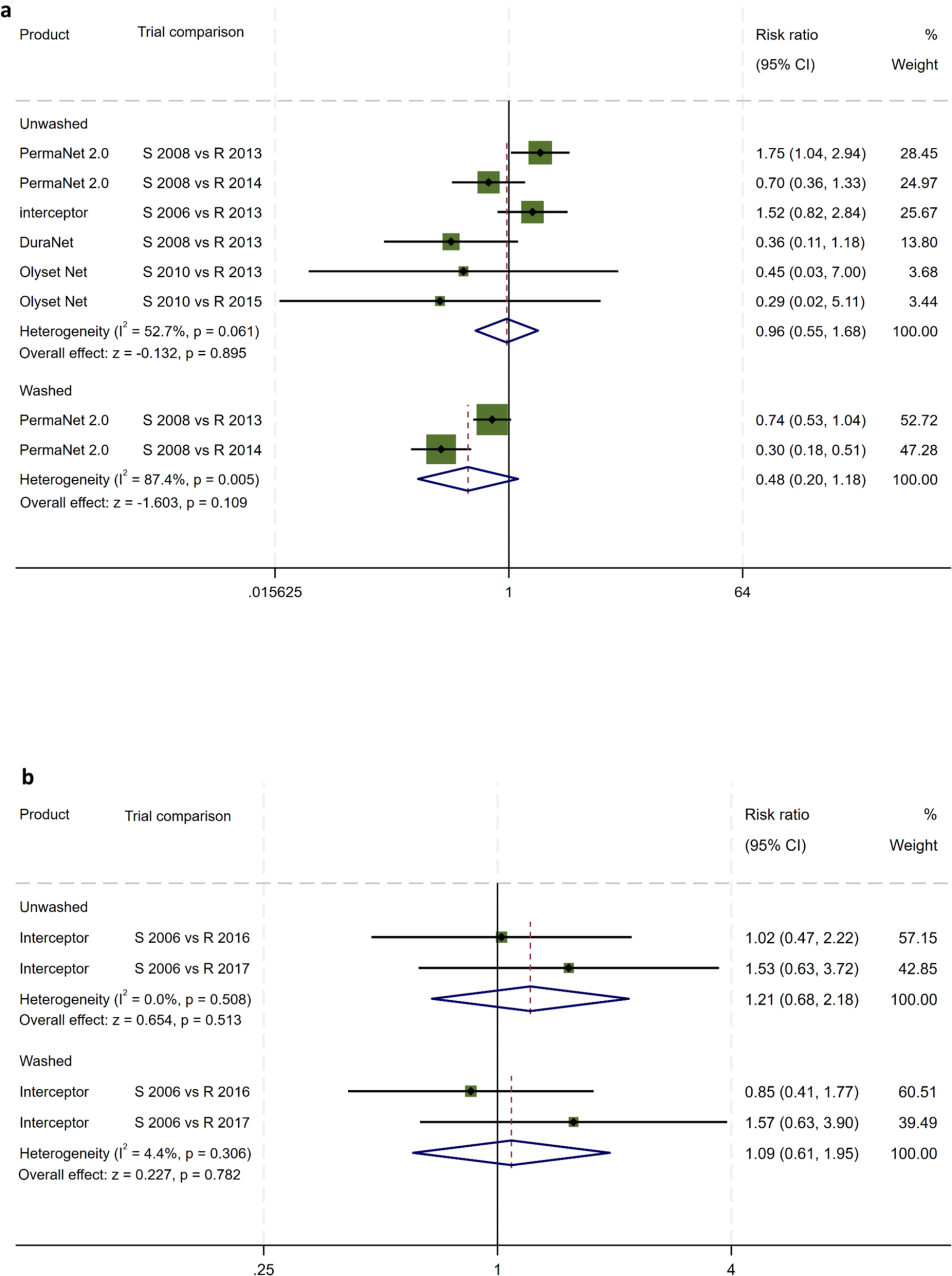
**a** Meta-analysis of the efficacy of washed and unwashed LLIN in hut trials done when vectors were susceptible and when resistant: *An. gambiae* s.l. blood-feeding risk ratio. **b** Meta-analysis of the efficacy of washed and unwashed LLIN in trials when vectors were susceptible and when resistant: *An. funestus* s.l. blood-feeding risk ratio

The meta-analysis pooled estimates of relative risks between the percentage of wild free-flying susceptible and resistant *An. gambiae* s.l. feeding on blood was not significantly different for either unwashed (RR = 1.01, 0.95–1.08) (*z* = 0.42, *P* = 0.677) or washed LLINs (RR = 0.97, 0.90–1.05) (*z* = 0.79, *P* = 0.429) (Fig. 4a).

The differences between pooled estimates of relative risks between the percentage of wild free-flying susceptible and resistant *An. funestus* s.l. that were feeding were also not significant for either unwashed (RR = 1.00, 0.86–1.17) (*z* = 0.05, *P* = 0.983) or washed (RR = 0.99, 0.86−1.15) (*z* = 0.08, *P* = 0.939) LLINs (Fig. 4b).

#### Logistic regression analysis of blood feeding

**Fig. 5:**
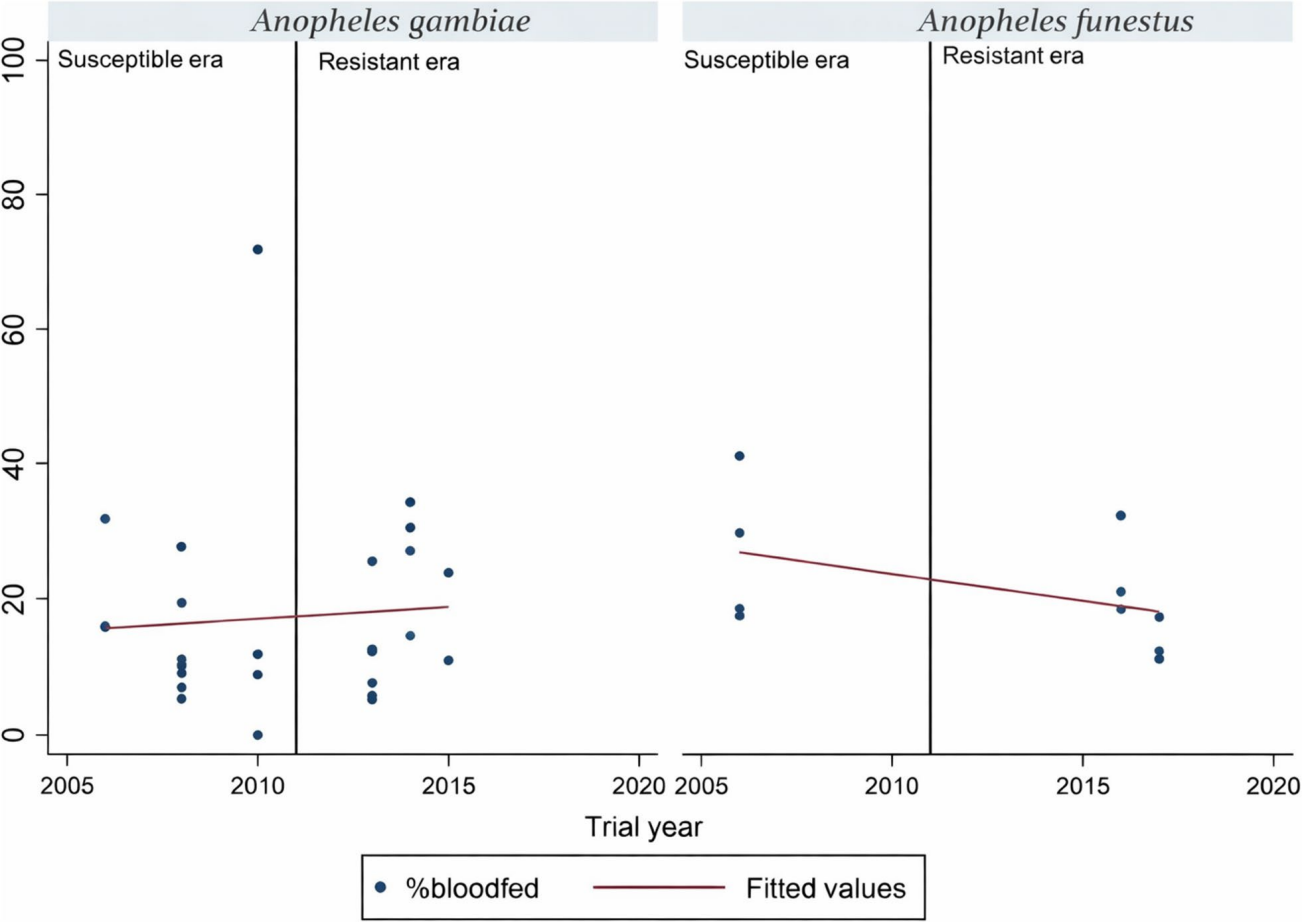
Percentage of wild free-flying *An. gambiae* s.l. and *An. funestus* s.l. feeding on blood during EHT. Each point denotes the percentage feeding on blood for each trial. The line graph shows best fit regression trend for the percentage feeding on blood in various trials. Solid vertical line demarcates trials when vectors were susceptible to those when vectors were resistant

The blood-feeding logistic regression analysis indicated that resistance was not associated with any change in the proportion of *An. gambiae* s.l. feeding on blood (*t* = 0.41, *P* = 0.686). With *An. funestus* s.l., there was not any trend evident between susceptibility and resistance, the proportions feeding on blood remaining around 25% (*z* = −1.48, *P* = 0.178) (Fig. 5).

### Mosquito exophily (numbers and proportions exiting the huts)

In the 2008 PermaNet 2.0 trial, when the vectors were susceptible, a naturally high proportion of *An. gambiae* were collected each morning from the verandah and window traps of huts with untreated nets. Insecticide-induced exophily of susceptible *An. gambiae* from huts with treated nets compared with huts with untreated nets was not significant, because most of the latter exited naturally each night from the huts (Table 5). However, in the 2013 trial when vectors were resistant, both the unwashed and washed PermaNet 2.0 LLINs recorded significantly more survival and exiting compared with when mosquitoes were susceptible (Table 5).

**Table 5:** Experimental hut trials: numbers entering, percentage deterrence, and percentage exiting for wild *An. gambiae* s.l. during PermaNet 2.0 hut trials conducted at Zeneti in 2008, 2013, and 2014

		Untreated net	PermaNet 2.0	PermaNet 2.0	CTN
Initial dose of deltamethrin (mg/m^2^)		0			25
Number of washes		0	Unwashed	20	3
Trial year					
2008	Total females entering	723	574	586	560
	Geometric mean females caught/night (95% CI)	13.4	10.6	10.9	10.4
	Percentage (%) deterrence	0^a,1^	20.6^a,1^	18.9^a,1^	22.5^a,1^
2013	Total females entering	445	289	548	452
	Geometric mean females caught/night (95% CI)	20.4	10.8	27.3	16.9
	Percentage (%) deterrence	0^a,1^	35.1^b,1^	0^a,1^	0^a,1^
2014	Total females entering	29	61	39	73
	Geometric mean females caught/night (95% CI)	1.7	1.7	1.5	1.9
	Percentage (%) deterrence	0^a,1^	0^a,1^	0^a,1^	0^a,1^
2008	Percentage (%) exiting	85.5^a,1^	85.5^a,1^	88.4^a,1^	92.0^b,1^
2013	Percentage (%) exiting	73.9^a,1^	94.1^b,1^	90.9^b,1^	89.2^b,1^
2014	Percentage (%) exiting	69^a,1^	88.5^b,1^	92.3^b,1^	65.8^c,1^

Percentage deterrence, exiting, and 95% CIs are back-transformed from values calculated using the blocked logistic regression model. Within each column, years not sharing a superscript number differ significantly using blocked logistic regression (*P* < 0.05). Within each row, treatments not sharing a superscript letter differ significantly using blocked logistic regression (*P* < 0.05). CI, confidence interval

In 2010, when vectors were susceptible, unwashed Olyset Net recorded induced deterrence of 80.9% and exiting of 30.8% (Table 6). In 2013 and 2015, in trials with resistant vectors, unwashed Olyset Net recorded significantly higher exiting rates (96.3% and 100%, respectively) relative to untreated control arms (68.8% and 81%, respectively) (Table 6).

**Table 6:** Experimental hut trials: number entering, percentage deterrence, and percentage exiting of wild *An. gambiae* s.l. during Olyset Net hut trials conducted at Zeneti in 2010, 2013, and 2015

		Untreated net	Olyset Net	Olyset Net	CTN
Initial dose of permethrin (mg/m^2^)		0			500
Number of washes		0	Unwashed	20	3
Trial year					
2010	Total females entering	68	13	43	107
	Geometric mean females caught/night	0.6	0.1	0.5	0.8
	Percentage (%) deterrence	0^a,1^	80.9^b,1^	36.8^c^	0^a,1^
2013	Total females entering	221	245		282
	Geometric mean females caught/night	3.4	4		4.7
	Percentage (%) deterrence	0^a,2^	0^a,2^	—	0^a,2^
2015	Total females entering	58	36		
	Geometric mean females caught/night	0.5	0.3		
	Percentage (%) deterrence	0^a,3^	37.9^b,1^	—	—
Trial year					
2010	Percentage (%) exiting	47.1^a,1^	30.8^b,1^	81.4^c^	88.8^c,1^
2013	Percentage (%) exiting	68.8^b,2^	96.3^b,2^	—	87.6^c,1^
2015	Percentage (%) exiting	81^c,3^	100^a,3^	—	—

Percentage deterrence, exiting, and 95% CI are back-transformed from values calculated by the blocked logistic regression model. Within each column, years not sharing a superscript number differ significantly using blocked logistic regression (*P* < 0.05). Within each row, treatments not sharing a superscript letter differ significantly using blocked logistic regression (*P* < 0.05). CI, confidence interval

Insecticide-induced exophily recorded with unwashed and washed Interceptor LN in 2006 and 2008 trials when vectors were susceptible ranged between 79% and 93%, but this was not significantly higher to rates recorded with untreated control. In 2013 when vectors were resistant, unwashed Interceptor LN recorded higher exophily (94.5%) than recorded with the untreated control (68.8%); hence, this hut trial showed evidence of enhanced insecticide induced exiting (Table 7).

**Table 7:** Experimental hut trials: number entering, percentage deterrence, and percentage exiting of wild *An. gambiae* s.l. during Interceptor hut trials conducted at Zeneti in 2006, 2008, and 2013

		Untreated net	Interceptor	Interceptor	CTN
Initial dose of alpha-cypermethrin (mg/m^2^)		0			40
Number of washes		0	Unwashed	20	3
Trial year					
2006	Total females entering	171	134	122	224
	Geometric Mean females caught/night (95% CI)	2.8	1.9	2.1	2.5
	Percentage (%) deterrence	0^a,1^	21.7^a,1^	28.7^a,1^	0^a^
2008	Total females entering	143	112	112	124
	Geometric mean females caught/night (95% CI)	2.6	2	1.8	3.4
	Percentage (%) deterrence	0^a,1^	21.4^b,1^	21.7^b,1^	13.10^c,1^
2013	Total females entering	221	255		282
	Geometric mean females caught/night (95% CI)	3.4	3.9		4.7
	Percentage (%) deterrence	0^a,2^	0^a,2^	—	0^a,2^
2006	Percentage (%) exiting	86^a,1^	86.6^a,1^	92.6^a,2^	92.8^a,1^
2008	Percentage (%) exiting	88.1^a,c,1^	79.5^b,1^	82.1^b,c,1^	89.5^a,c,1^
2013	Percentage (%) exiting	68.8^a,2^	94.5^b,2^	—	87.6^c,2^

Percentage deterrence, exiting, and 95% CIs are back-transformed from values calculated by the blocked logistic regression model. Within each column, years not sharing a superscript number differ significantly using blocked logistic regression (*P* < 0.05). Within each row, treatments not sharing a superscript letter differ significantly using blocked logistic regression (*P* < 0.05)

There were no significant differences in exiting rate of *An. funestus* from the huts with untreated nets as compared with that recorded from the huts with treated nets (unwashed or 20-times-washed Olyset Net) in 2006 and 2008, thus no evidence of enhanced insecticide induced exiting rate of *An. funestus* (RR = 0.99, 0.86–1.15) (*z* = 0.08, *P* = 0.939).

#### Meta-analysis pooled estimate of mosquito exiting

Results from meta-analysis also showed that with unwashed LLINs different levels of exophily were recorded between susceptible and resistant *An. gambiae* s.l. (RR = 0.91, 0.85–0.97; *z* = 2.79, *P* = 0.005) indicating resistant *An. gambiae* s.l. were more likely to exit the huts with the unwashed LLINs as compared with susceptible *An. gambiae* s.l.; the increase in exophily of the resistant mosquitoes was significant with the unwashed LLINs (Electronic Supplementary Material [ESM] 1a). However, with washed LLINs similar levels of exiting were recorded (RR = 0.98, 0.94–1.01; *z* = 1.45, *P* = 0.148) (ESM 1a).

#### Meta-analysis pooled estimate of exophily

Resistant *An. funestus* s.l. with unwashed and 20-times-washed LLIN were 1.21 (1.10–1.33) and 1.25 (1.00–1.56) less likely to exit as compared with susceptible *An. funestus* s.l. (*z* = 3.9, *P* = 0.001) and (*z* = 1.98, *P* = 0.047) with unwashed and washed LLINs, respectively (ESM 1b).

#### Logistic regression analysis of exophily

Regression analysis of trials showed that although there was increase in exophily with resistant *An. gambiae* s.l. the trend was not significant (*t* = −0.30, *P* = 0.764). With *An. funestus* s.l. the decreasing trend to exophily with resistant mosquitoes as compared with susceptible mosquitoes was significant; hence, resistance was significantly associated with changes in LLIN-induced exophily of wild free-flying *An. funestus* s.l. (*t* = 3.19, *P* = 0.013) (ESM file 2).

### Mosquito deterrence (numbers caught inside the huts)

The numbers and proportions caught in the experimental huts are shown in Tables 5–7. The geometric mean number (GMN) of *An. gambiae* collected per hut per night during PermaNet 2.0 trials ranged between 10–13, 11–27, and 1.5–1.9 in 2008, 2013, and 2014, respectively (Table 5). The GMN of *An. gambiae* s.l. collected during the Olyset Net trials ranged between 0.6–0.8, 3–5, and 0.3–0.5 in 2010, 2013, and 2015 (Table 6). The GMN collected during the Interceptor LN trials ranged between 2–2.8, 2–3.4, and 3.4–4.7 in 2006, 2008, and 2013 (Table 7). The PermaNet 2.0 and Olyset Net trials in 2014 and 2015 were conducted during the short rainy season; therefore, fewer numbers of *An. gambiae* were collected, with the GMN ranging between 1.5–1.7 and 0.3–0.5, respectively.

What is the evidence for LLIN-induced deterrence compared with numbers collected in huts with untreated control nets? There were significant reductions in the numbers of mosquitoes collected from the huts with pyrethroid-treated nets in the 2013 PermaNet 2.0 LN trial, in 2010 and 2015 Olyset Net LN trials, and in the 2008 Interceptor LN trial. This indicates the advantage of always using LLIN, even when holed.

There were sometimes small differences in the number of mosquitoes collected from the huts with treated LLIN either unwashed or 20 times washed. This was the original purpose of WHO putting each brand through WHOPES and WHOPQT evaluation, to drive up and then maintain quality standards.

#### Meta-analysis pooled estimate of deterrence

Results from meta-analysis also showed that similar levels of deterrence were recorded between susceptible and resistant *An. gambiae* s.l. against both unwashed (*z* = 1.47, *P* = 0.141) and washed (*z* = 0.09, *P* = 0.951) LLINs (ESM 3a). Conversely, there was significant increase in deterrence with resistant *An. funestus* s.l. compared with susceptible *An. funestus* s.l. against both unwashed (*z* = 2.72, *P* = 0.006) and washed (*z* = 7.66, *P* = 0.001) LLINs (ESM 3b).

#### Logistic regression analysis of deterrence

The regression analysis showed that the transition from susceptibility to resistance was not associated with any change in LLIN-induced deterrence of flying *An. gambiae* s.l (*t* = −1.35, *P* = 0.186) or *An. funestus* s.l. (*t* = 1.85, *P* = 0.101) (ESM 4).

## Discussion

The study assessed the impact of insecticide resistance on the efficacy of pyrethroid LLINs in a series of ten LLIN evaluations in experimental hut trials in Northeastern Tanzania between 2006 and 2017. The early hut trials were conducted when *An. gambiae* s.l. and *An. funestus* s.l. were fully susceptible to pyrethroids; later hut trials were conducted after the development of pyrethroid resistance in the two vectors. The fixed hut location and changing vector resistance status provided a unique opportunity to study the changing effectiveness of standard LLIN and their capacity to control mosquitoes at a site that had vector population that had become increasingly resistant over the decade.

The meta-analysis of trials, before and after resistance, on the same brands of net was a novel approach showing that mortality risk ratio of susceptible versus resistant free-flying *An. gambiae* s.l. was 6.7 times higher than with unwashed LLIN and 5.2 times higher than with the 20-times-washed LLIN tested against a variety of pyrethroids, net brands, and textile materials. The meta-analysis on free-flying *An. funestus* s.l. showed mortality risk ratios were 3.3 and 2.6 times higher on the same brand of nets for susceptible compared with resistant mosquitoes. The meta-analysis confirmed loss of efficacy of pyrethroid LLINs to control pyrethroid resistant vectors.

It was shown by regression analysis that significantly fewer resistant *An. gambiae* s.l. and *An. funestus* s.l. mosquitoes were killed by LLINs compared with susceptible mosquitoes over several years in succession.

Reductions in efficacy of insecticidal interventions, due to resistance, have been reported in several countries in Africa, including Benin and Burkina Faso in West Africa [25, 47], Uganda [48], and Tanzania in East Africa [49]. None of these studies have investigated, at a single location, the impact of pyrethroid resistance on household effectiveness (i.e., EHT) of LLIN in such detail over such a prolonged period [50–52].

The Tanzanian study provides evidence of association between pyrethroid resistance and LLIN efficacy, whereby resistant *An. gambiae* s.l. and *An. funestus* s.l. mosquitoes were more likely to survive and exit the huts. The meta-analysis comparing the induced exophily showed greater rate of exiting with resistant versus susceptible *An. gambiae* s.l. on the unwashed and 20-times-washed LLINs.

The meta-analyses found little evidence for major differences in the proportion blood feeding of resistant compared with susceptible *An. gambiae* s.l. or *An. funestus* s.l. The meta-analyses confirmed that any difference was nonsignificant for either species, whether with unwashed or 20-times-washed LLIN. Personal protection due to pyrethroid irritability of the net barrier would be expected to continue after resistance has evolved until individual nets become highly holed.

Although insecticide resistance had a negative entomological effect on LLIN efficacy, the study did not encompass an analysis of malaria epidemiology or vector EIR, and therefore, the EHT data are inferential rather than conclusive and should not be interpreted as failure of standard LLIN to control malaria. The interaction between resistance and changes to mosquito physiology and behavior is complex and may not lead to LLIN failure [53–56]. In this study, resistant Anophelines were significantly more likely to exit the huts as compared with susceptible Anophelines. The increase in exiting behavior in resistant *An. gambiae* s.l. combined with stasis in feeding behavior led to resistance-induced LLINs avoidance behavior [57]. Thus, although mosquitoes might not be killed by the standard LLIN owing to resistance, the change in behavior induced by pyrethroid may lessen the capacity for mosquitoes to continue probing through the net and feed on a human occupying a slightly torn net. This, together with other physiological factors, such as the reduction in the rate of malaria oocyst development in pyrethroid-exposed resistant mosquitoes [58], the possibility of delayed mortality beyond the 24 h standard holding interval when exposed to pyrethroids [59], and resistant mosquitoes being less fit compared with susceptible ones [59, 60], may combine and lead to reduced lifespan and fewer older infective mosquitoes. These resistance-induced behavioural and physiological factors may operate additively to reduce the negative epidemiological impact that resistance may otherwise have, and lead to continued efficacy of LLIN in controlling malaria mosquitoes in areas with resistant mosquitoes, as reported in epidemiological studies that show LLIN users are protected against malaria compared with non-LLIN users [21, 53, 54, 56, 61–63].

However, in the 2018 factorial trial of PBO–pyrethroid nets, there was clear epidemiological evidence that PBO–pyrethroid LLIN were superior to pyrethroid-only standard LLIN at preventing malaria in Tanzania [64]. The more recent four-arm cluster randomized controlled trials (cRCT) that have evaluated new-generation PBO–pyrethroid or dual-AI LLINs in locations of high resistance in East and West Africa reported that new-generation LLIN showed improved entomological and epidemiological effect compared with the standard pyrethroid-only LLIN [65, 66], which failed when they acquired holes and the net barrier is breached [23]. High resistance not only undermines entomological efficacy; it may undermine any epidemiological argument for issuing standard LLIN as opposed to PBO or dual-AI LLIN. This highlights the need for more new insecticide classes with novel modes of action that can improve management of resistant mosquitoes, and new strategies for effective, sustainable resistance management.

During this longitudinal study, resistance mechanisms were identified using WHO susceptibility tests and molecular *kdr* screening with limited metabolic confirmation from PBO assays. More advanced biochemical or transcriptomic profiling could have provided stronger causal insights had they been available at the time. More recently in Tanzania (2022), Matowo and Weetman, using transcriptomic analysis, found multiple P450 genes overexpressed, including CYP6M2, CYP6Z3, CYP6P3, CYP6P4, CYP6AA1, and CYP9K1 in *An. gambiae* and CYP6N1, CYP6M7, CYP6M1, and CYP6Z1 in *An. funestus* [67]. The same authors have also shown a distinct taxon with a unique profile of genetic diversity and appears restricted to Muheza referred to as the Pwani molecular form [68].

The most powerful design to evaluate pyrethroid LLIN against anthropophilic species such as *An. gambiae* s.l. and *An. funestus* is the cluster randomized controlled trial (cRCT), as it can have a “mass population killing effect” on mosquito populations when taken to total household coverage in village communities (WHO 2018). Experimental hut trials (EHT) measure effectiveness at the household level and may miss measuring vector impact outside the home, which a cRCT design would not do. However, PBO-LLIN cRCT over 3 years have already been completed in Tanzania [64, 65], whereas longitudinal series of EHTs, which traverse the susceptible-resistance divide over the decade, have not been done before. This is a unique difference from cRCT, clearly; there is a need for both cRCT and longitudinal EHT trials to determine both area and household effects on malaria vector control and management of insecticide resistance.

## Conclusions

The reduced efficacy of pyrethroid-treated nets (LLINs) on entomological indices in an area with pyrethroid resistant *An*. *gambiae* s.l. and *An. funestus* s.l. confirms that, in Tanzania, resistance undermines the effectiveness of pyrethroid LLINs. To safeguard insecticide-based vector control, new insecticide classes with novel modes of action need to be scaled up to achieve the targets of the WHO Global Technical Strategy for Malaria 2016–2030.

## Supplementary Information

Supplementary Material 1: Supplementary file 1a. Meta-analysis of the efficacy of washed and unwashed LLIN in trials when vectors were susceptible and when resistant: *An. gambiae* s.l. exophily risk ratios. 1b. Meta-analysis of the efficacy of washed and unwashed LLIN in hut trials when vectors were susceptible and when resistant: *An. funestus* s.l. exophily risk ratios

Supplementary Material 2: Supplementary file 2. Percentage exiting of wild, free-flying *An. gambiae* s.l. and *An. funestus* s.l. from experimental huts during trials before and after resistance. The line graph shows the best fit regression trend line for the percentage exiting in various trials. Solid vertical lines demarcate trials when vectors were susceptible and when resistant

Supplementary Material 3: Supplementary file 3a. Meta-analysis of the efficacy of unwashed and washed LLIN in trials when vectors were susceptible and when resistant: *An. gambiae* s.l. deterrence risk ratios. Supplementary file 3b. Meta-analysis of the efficacy of unwashed and washed LLIN in hut trials when vectors were susceptible and when resistant: *An. funestus* s.l. deterrence risk ratios

Supplementary Material 4: Supplementary file 4. Percentage deterrence of wild free-flying *An. gambiae* s.l. and *An. funestus* s.l. Each point denotes the number entering relative to the control for each trial. The line graph shows the best fit regression trend line for the percentage exiting in various trials. The vertical lines demarcate trials done when vectors were susceptible to those done when vectors were resistant

## Data Availability

Data supporting the main conclusions of this study are included in the manuscript.
