# Supplementary figures and images for: Efficacy of long-lasting insecticidal nets before and after the selection of pyrethroid-resistant Anopheles gambiae s.l. and Anopheles funestus s.l. in northeastern Tanzania: A longitudinal meta-analysis of experimental hut trials at a single location
Source: Parasit Vectors. 2026 Feb 25;19:141. doi: 10.1186/s13071-026-07294-4 (PMC13041117; doi:10.1186/s13071-026-07294-4)

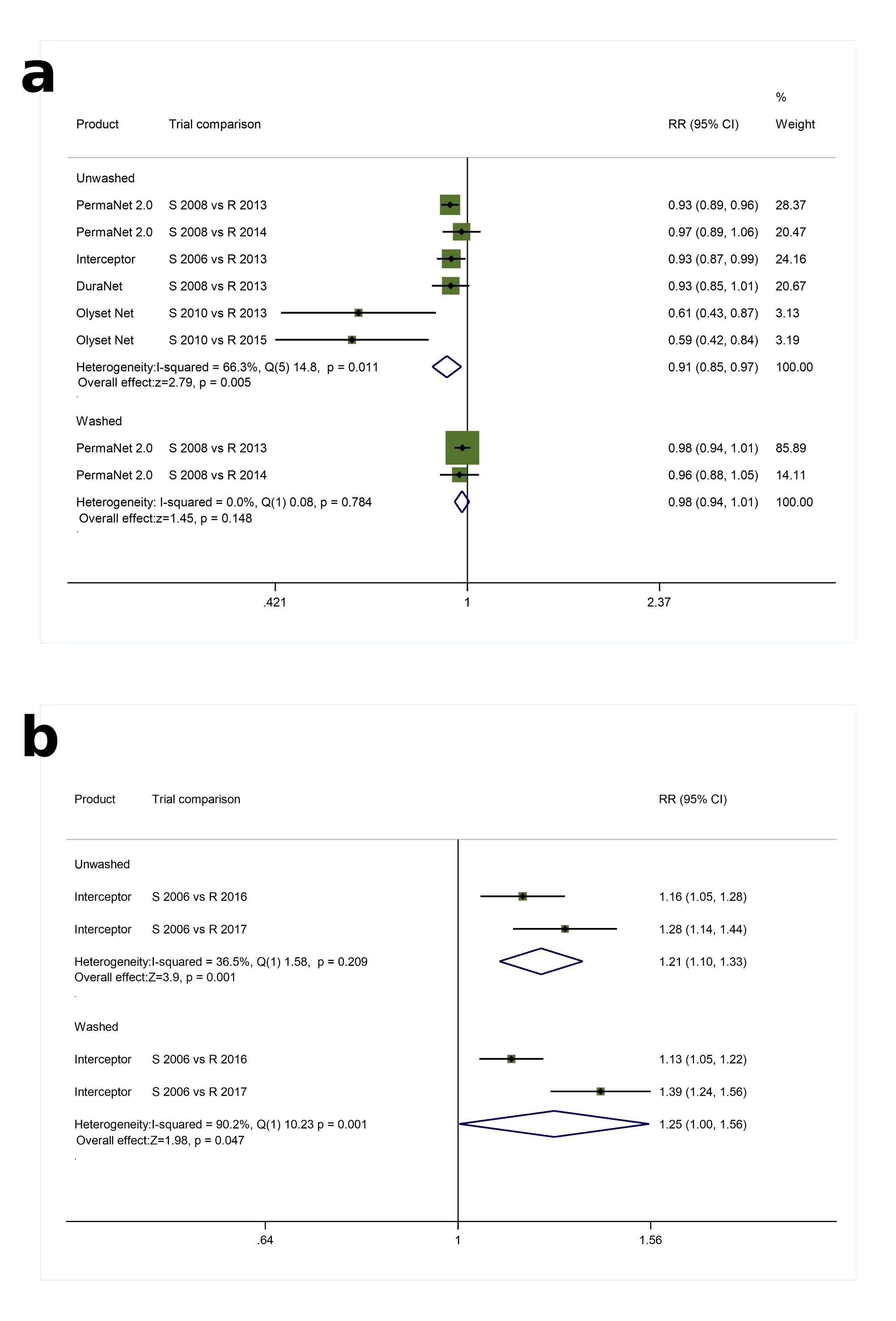

Supplement: Supplementary file 1 — Supplementary Material 1: Supplementary file 1a. Meta-analysis of the efficacy of washed and unwashed LLIN in trials when vectors were susceptible and when resistant: An. gambiae s.l. exophily risk ratios. 1b. Meta-analysis of the efficacy of washed and unwashed LLIN in hut trials when vectors were susceptible and when resistant: An. funestus s.l. exophily risk ratios [file 13071_2026_7294_MOESM1_ESM.jpg]

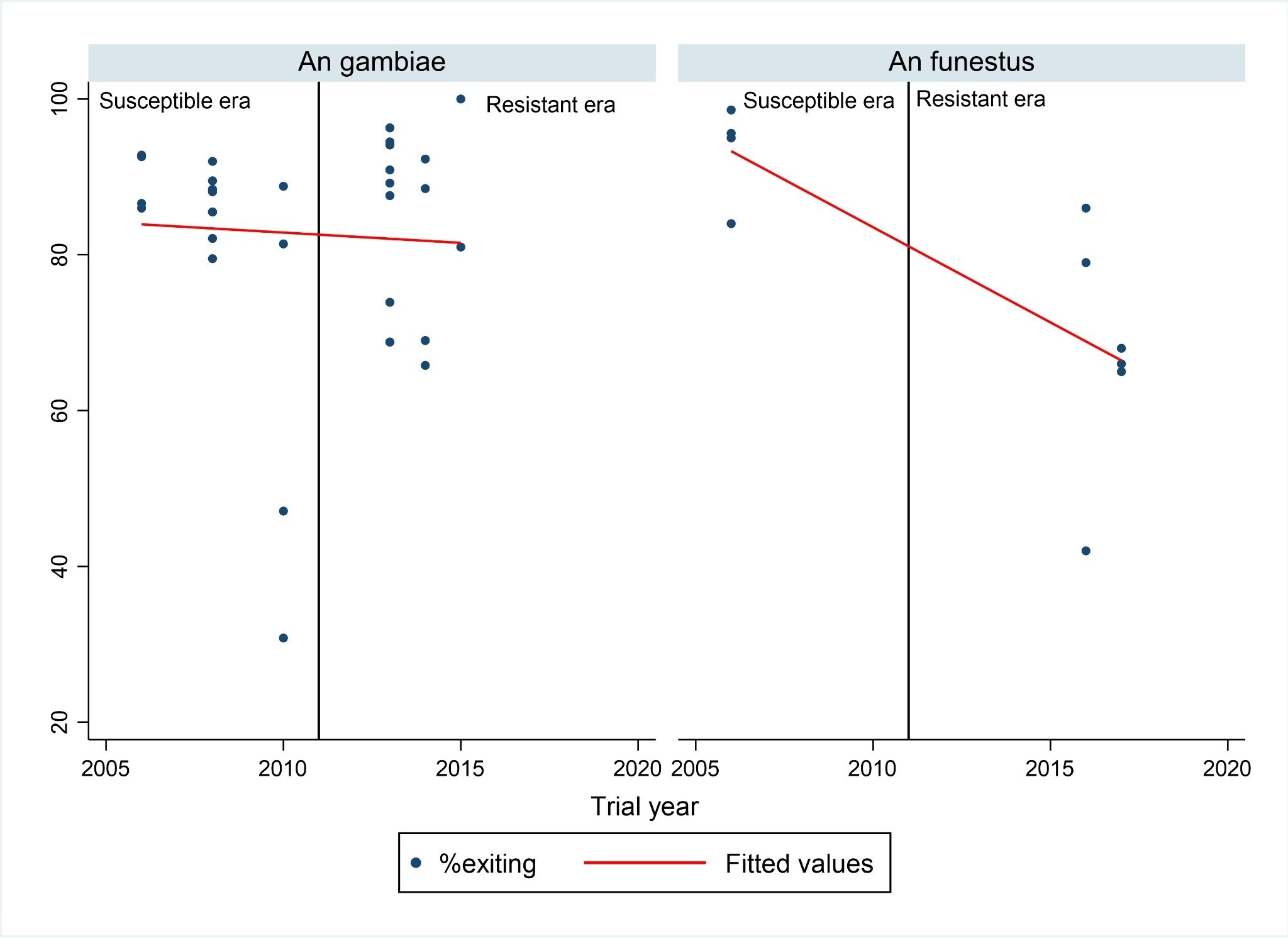

Supplement: Supplementary file 2 — Supplementary Material 2: Supplementary file 2. Percentage exiting of wild, free-flying An. gambiae s.l. and An. funestus s.l. from experimental huts during trials before and after resistance. The line graph shows the best fit regression trend line for the percentage exiting in various trials. Solid vertical lines demarcate trials when vectors were susceptible and when resistant [file 13071_2026_7294_MOESM2_ESM.jpg]

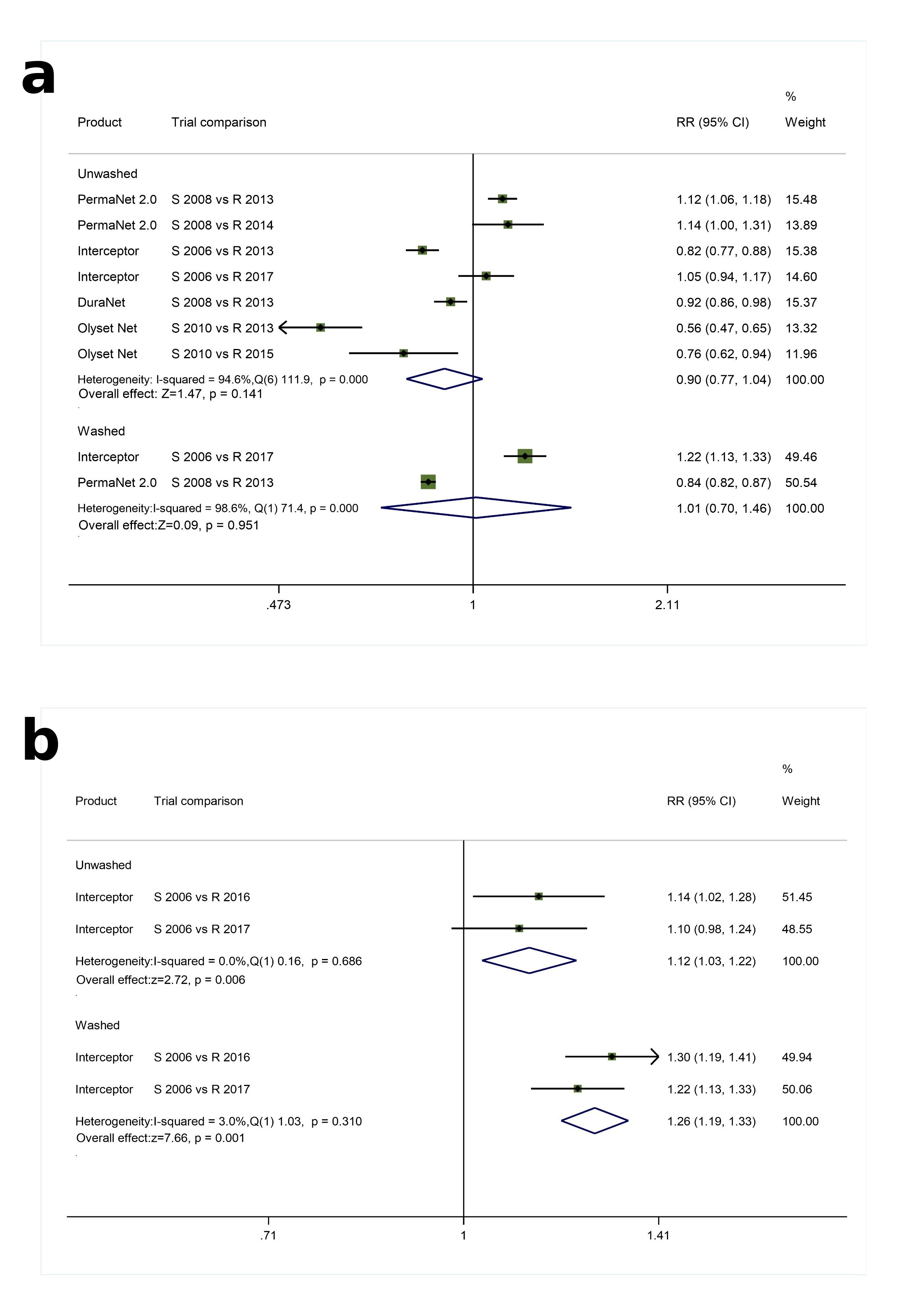

Supplement: Supplementary file 3 — Supplementary Material 3: Supplementary file 3a. Meta-analysis of the efficacy of unwashed and washed LLIN in trials when vectors were susceptible and when resistant: An. gambiae s.l. deterrence risk ratios. Supplementary file 3b. Meta-analysis of the efficacy of unwashed and washed LLIN in hut trials when vectors were susceptible and when resistant: An. funestus s.l. deterrence risk ratios [file 13071_2026_7294_MOESM3_ESM.jpg]

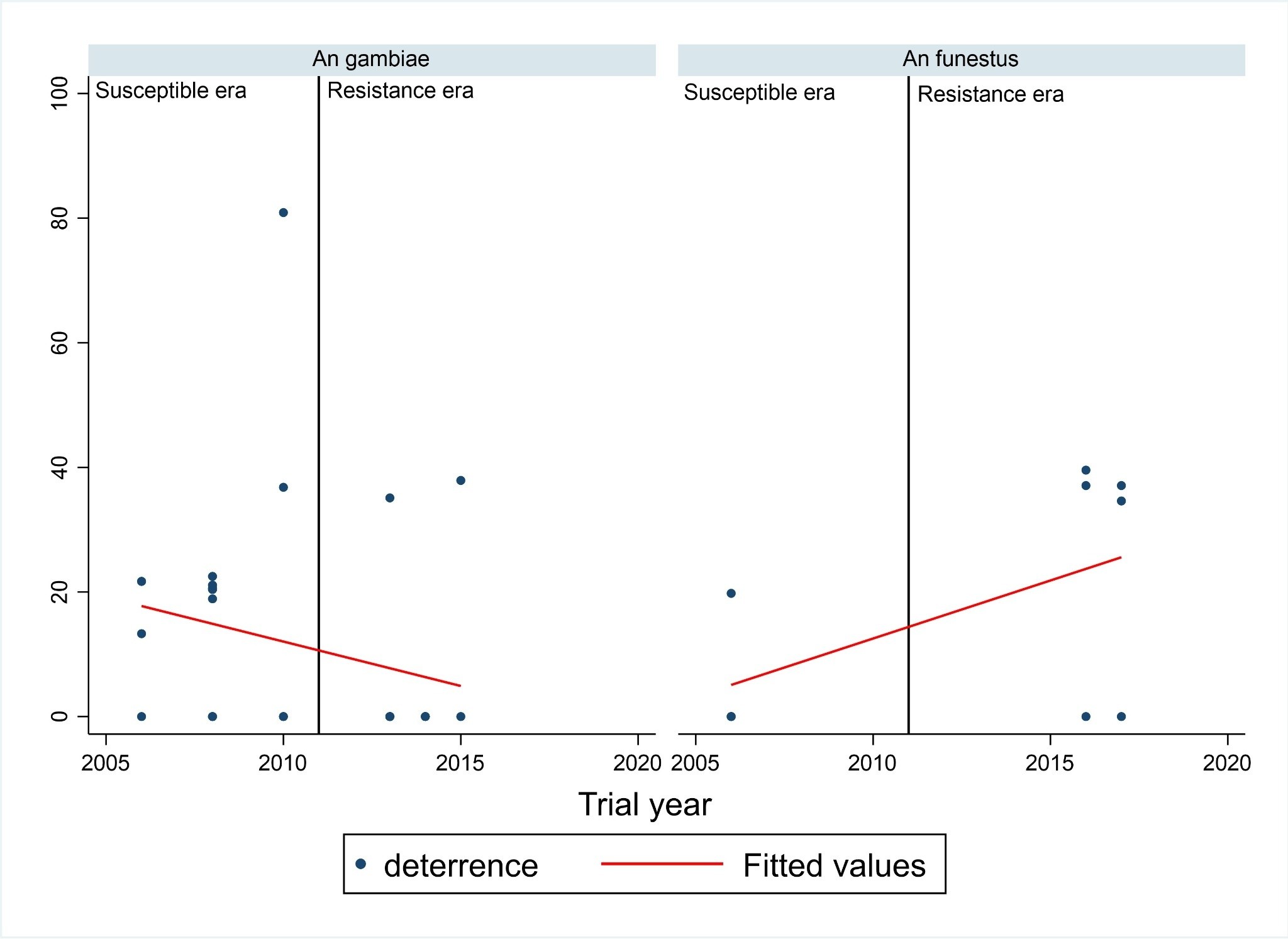

Supplement: Supplementary file 4 — Supplementary Material 4: Supplementary file 4. Percentage deterrence of wild free-flying An. gambiae s.l. and An. funestus s.l. Each point denotes the number entering relative to the control for each trial. The line graph shows the best fit regression trend line for the percentage exiting in various trials. The vertical lines demarcate trials done when vectors were susceptible to those done when vectors were resistant [file 13071_2026_7294_MOESM4_ESM.jpg]
